# Directed or Random? Student Reasoning About Diffusion Across Physiological Contexts

**DOI:** 10.51894/001c.164398

**Published:** 2026-08-01

**Authors:** Zach Kam, Maya Shah, Jasmine Parker, Issac Gollapalli

**Affiliations:** 1 College of Osteopathic Medicine, Michigan State University

## 46

### Background

Random molecular motion underlies diffusion, chemical reactions, and gas exchange, yet many students explain diffusion primarily as particles moving “from high to low concentration.” Rather than treating this as a misconception, we adopt a dynamic systems perspective informed by Knowledge-in-Pieces and Resources frameworks to examine how students coordinate knowledge resources when reasoning about diffusion.

### Methods

We administered three parallel diffusion questions (animal, plant, and nonliving contexts) to 197 students enrolled in an introductory human physiology course at a large R1 public university. Students predicted the location of a labeled oxygen molecule before and after equilibrium and explained their reasoning. Using knowledge analysis with team-based coding (90% interrater reliability), we identified common knowledge resources and patterns in their coordination. Multinomial regression assessed effects of context and GPA on reasoning type.

### Results

Fourteen common knowledge resources were identified and organized into six reasoning patterns. The most prevalent pattern was “High to Low” reasoning (51%), in which students relied on concentration gradients to explain molecular behavior. Twenty-one percent coordinated gradient reasoning with random motion after equilibrium, while only 2% used exclusively random motion reasoning. Question context did not significantly impact reasoning type. Across GPA levels, “High to Low” reasoning predominated, although higher-GPA students were somewhat more likely to coordinate gradient and random motion resources.

### Conclusions

Students’ reliance on “High to Low” reasoning reflects its strong cueing priority in diffusion contexts. Instruction should therefore focus on helping students coordinate productive gradient reasoning with molecular-level random motion rather than replacing existing ideas. A resources-based approach may better support mechanistic understanding of diffusion.

